# Prediction of cognitive outcome and progression to dementia using ω6‐PUFA/ω3‐PUFA ratio

**DOI:** 10.1002/alz.71590

**Published:** 2026-06-10

**Authors:** Víctor Andrade, Luca Kleineidam, Holger Wagner‐Thelen, Tommaso Ballarini, Rafael Campos‐Martin, Pamela Martino‐Adami, Kumar Parijat Tripathi, Sophie Guyonnet, Bruno Vellas, Martin Scherer, Wolfgang Maier, Michael Pentzek, Matthias Schmid, Steffi Riedel‐Heller, Siegfried Weyerer, Horst Bickel, Birgitt Wiese, Sarah Egert, Michael Wagner, Alfredo Ramírez

**Affiliations:** ^1^ Division of Neurogenetics and Molecular Psychiatry Department of Psychiatry and Psychotherapy Medical faculty University of Cologne Cologne Germany; ^2^ Department of Cognitive Disorders and Old Age Psychiatry University Hospital Bonn Bonn Germany; ^3^ German Center for Neurodegenerative Diseases (DZNE) Bonn Germany; ^4^ Centre d'Epidémiologie et de Recherche en santé des Populations de Toulouse Toulouse France; ^5^ Department of Geriatric Internal Medicine Toulouse University Hospital Toulouse France; ^6^ Department of Primary Medical Care Center for Psychosocial Medicine University Medical Center Hamburg‐Eppendorf Hamburg Germany; ^7^ Medical Faculty Institute of General Practice Primary Care Research University of Duisburg‐Essen Essen North Rhine‐Westphalia Germany; ^8^ Department of Medical Biometry Informatics and Epidemiology University Hospital Bonn Bonn Germany; ^9^ Institute of Social Medicine Occupational Health and Public Health University of Leipzig Leipzig Germany; ^10^ Central Institute of Mental Health Medical Faculty Mannheim Heidelberg University Mannheim Germany; ^11^ Department of Psychiatry Technical University Munich Germany; ^12^ Institute for General Practice Hannover Medical School Hannover Lower Saxony Germany; ^13^ Institute of Nutritional and Food Sciences Nutritional Physiology University of Bonn Bonn Germany; ^14^ Glenn Biggs Institute for Alzheimer's & Neurodegenerative Diseases University of Texas Health Sciences Center San Antonio Texas USA; ^15^ Cologne Excellence Cluster on Cellular Stress Responses in Aging‐Associated Disease (CECAD) University of Cologne Cologne Germany

**Keywords:** Alzheimer's disease, cognition, polyunsaturated fatty acids, prevalent neurological disorders, ω3‐PUFA, ω6‐PUFA

## Abstract

**INTRODUCTION:**

The polyunsaturated fatty acids (PUFAs) omega‐6 (ω6) and omega‐3 (ω3) are linked to cognitive performance and Alzheimer's‐type dementia (DAT), but ω3‐PUFA supplementation offers limited benefits. We propose that the ω6‐PUFA/ω3‐PUFA ratio better explains cognitive decline and DAT risk.

**METHODS:**

PUFA profiles were examined in the AgeCoDe cohort (*n* = 3327) and MAPT trial (*n*  = 1679). Cox and linear mixed models evaluated associations of individual PUFAs and the ω6‐PUFA/ω3‐PUFA ratio with DAT progression and cognitive decline. Mendelian randomization (MR) assessed genetic causal effects. The effect of ω3‐PUFA on ω6‐PUFA levels was analyzed.

**RESULTS:**

Higher ω6‐PUFA/ω3‐PUFA ratio increased DAT risk beyond ω3‐PUFA levels alone. A baseline high (detrimental) ratio predicted faster cognitive decline, whereas longitudinal improvements slowed decline. MR supported a genetic non‐causal link. Higher ω3‐PUFA levels correlated with lower ω6‐PUFA species.

**DISCUSSION:**

The ω6‐PUFA/ω3‐PUFA ratio better predicts cognitive decline and DAT progression than individual PUFAs, suggesting that dietary adjustments may help prevent dementia.

## Highlights

- Omega 6 polyunsaturated fatty acid (ω6‐PUFA)/ω3‐PUFA ratio predicted cognitive decline and dementia better than individual PUFAs.
- Higher ω3‐PUFA levels were associated with reduced ω6‐PUFA species.
- Genetic analysis supported a non‐causal effect of PUFAs on progression to dementia.
- Active reduction of the ω6‐PUFA/ω3‐PUFA ratio led to better cognitive outcomes over time.
- ω6‐PUFA/ω3‐PUFA ratio balance emerges as a modifiable target for dementia prevention.

## 1 BACKGROUND

Dementia is a diverse group of neurodegenerative conditions, with Alzheimer's disease (AD) being the most common, making up 60%–80% of all cases.^1^ As a multifactorial syndrome, dementia results from the interaction of various modifiable and non‐modifiable factors. Of note, modifiable factors provide an important opportunity for prevention, with estimates indicating that up to 40% of dementia cases could be prevented or delayed through targeted interventions.^2^

Among modifiable risks, nutrition has emerged as a crucial factor in long‐term cognitive health. However, Western dietary patterns, often characterized by high calorie intake, increased saturated fats, and an imbalanced fatty acid profile (with respect to omega‐6 polyunsaturated fatty acid [ω6‐PUFA] and omega‐3 polyunsaturated fatty acid [ω3‐PUFA] levels), have been associated consistently with cardiometabolic disorders such as type 2 diabetes and cardiovascular disease, both of which increase the risk of dementia.^3^, ^4^ Western diets (WDs) are notably high in ω6‐PUFA and relatively low in ω3‐PUFA.^5^ Observational studies have reported protective associations of ω3‐PUFA species with cognitive resilience and a reduced risk of dementia, especially eicosapentaenoic acid (EPA) and docosahexaenoic acid (DHA).^3^, ^6^ However, randomized trials supplementing ω3‐PUFA have yielded inconsistent results,^7^, ^8^, ^9^, ^10^ indicating that additional biological mechanisms underlying the neuroprotective effect of ω3‐PUFA remain poorly understood.

In this context, the imbalance between ω6‐PUFA and ω3‐PUFA may act as the missing link related to PUFAs and the risk of chronic diseases, including dementia.^9^, ^11^, ^12^ Optimal neurological function is believed to be supported by a ω6‐PUFA/ω3‐PUFA ratio of ≈1:1 to 3:1, yet modern diets often have ratios of 10:1 to 20:1 or higher.^5^, ^13^ Supporting this, nutritional interventions mimicking the Mediterranean diet (MeDi), which emphasizes higher intake of ω3‐PUFA and lower ω6‐PUFA, have consistently been linked to a reduced risk of dementia.^6^, ^11^ These fatty acids have opposing roles in inflammation and neuronal health: ω3‐PUFA promote anti‐inflammatory and neuroprotective effects, whereas ω6‐PUFAs, especially arachidonic acid (ARA), are precursors to pro‐inflammatory mediators that may increase dementia risk.^4^, ^14^ Because both types utilize the same enzymatic pathways, excessive intake of ω6‐PUFA can hinder ω3‐PUFA metabolism through competition, reducing their benefits even with adequate consumption.^15^ However, direct evidence connecting the ω6‐PUFA/ω3‐PUFA ratio to incident dementia remains limited.

We therefore hypothesized that the neuroprotective effects of ω3‐PUFA depend critically on the balance between ω6‐PUFA and ω3‐PUFA. To test this hypothesis, we evaluated the role of the ω6‐PUFA/ω3‐PUFA ratio in the risk of progression to Alzheimer's‐type dementia (DAT) and cognitive decline in non‐demented adults, using two independent longitudinal cohorts: the German Study on Aging, Cognition, and Dementia (AgeCoDe) and the Multidomain Alzheimer Preventive Trial (MAPT). We also assessed whether common genetic variants modulating ARA/EPA, and specifically within the fatty acid desaturase locus (key regulators of PUFA metabolism) contribute to DAT risk. Finally, we examined whether ω3‐PUFA levels influence blood levels of ω6‐PUFA species, providing mechanistic insights into how PUFA balance affects dementia‐related trajectories.

**RESEARCH IN CONTEXT**

1. **Systematic review**: We searched PubMed from July 2020 to June 2025 for studies on polyunsaturated fatty acid (PUFA), cognition, and dementia, focusing on the omega 6 (ω6)‐PUFA/ω3‐PUFA ratio, intervention trials with ω3‐PUFA supplementation, and research on the genetics of PUFA and Alzheimer's‐type dementia (DAT). Previous reports have shown mixed cognitive benefits from ω3‐PUFA supplementation and limited evidence on the role of ω6‐PUFA in dementia risk.
2. **Interpretation**: Using two large, independent cohorts, we demonstrated that the ω6‐PUFA/ω3‐PUFA ratio is a more accurate predictor of cognitive decline and DAT progression than individual PUFAs. Genetic analyses indicated a non‐causal contribution. Finally, we found that higher ω3‐PUFA levels influence ω6‐PUFA species.
3. **Future directions**: Future studies should include dietary interventions that focus on the ω6‐PUFA/ω3‐PUFA ratio and identify optimal intake levels. New evidence is needed to elucidate the missing molecular mechanisms underlying processes such as neuroinflammation. The ω6‐PUFA/ω3‐PUFA ratio appears to be a promising biomarker for monitoring dietary compliance and a potential strategy for DAT prevention.

## 2 METHODS

### 2.1 Datasets and study design

**FIGURE 1:**
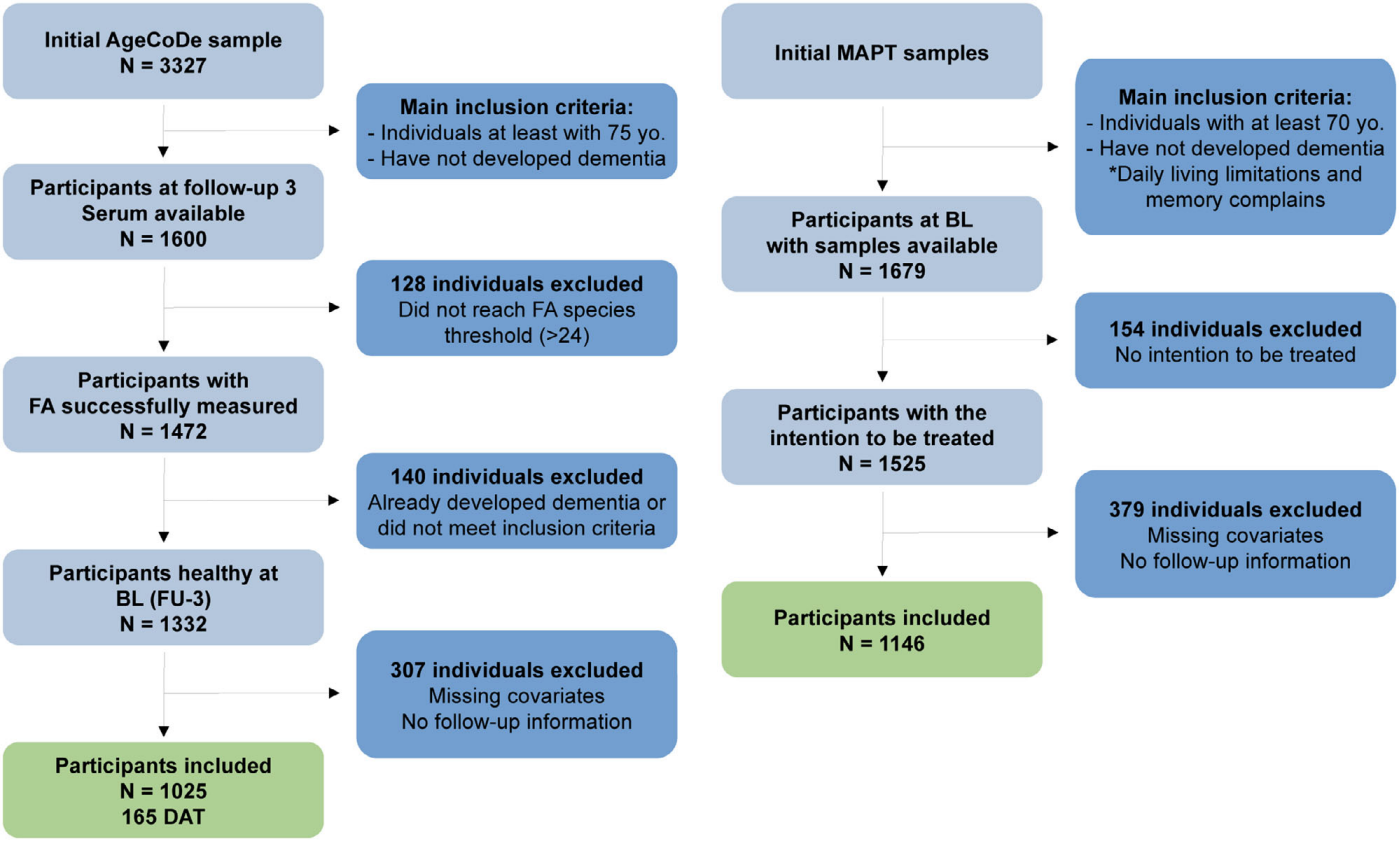
AgeCoDe and MAPT datasets pre‐processing for subsequent analyses. *MAPT full detail inclusion criteria can be found at Supplementary Information 2.1. yo, years old; FA, fatty acids; BL, baseline; FU, follow‐up.

This study integrates data from the AgeCoDe^16^, ^17^ and MAPT^18^, ^19^ cohorts, two well‐established longitudinal studies of aging and cognitive health. Sample availability and study design workflows are presented in Figure 1. Cohort demographics are provided in Table S1.

*AgeCoDe* *dataset*. AgeCoDe is a primary care longitudinal cohort initiated in six German cities between 2003 and 2004.^16^ Eligible participants were ≥75 years of age, free of dementia according to general practitioners’ (GPs) assessments, and had visited their GP within the past 12 months. Exclusion criteria included nursing home residence, terminal illness that the GP would deem fatal within 3 months, insufficient facility in German, deafness or blindness, inability to provide consent, or irregular contact with the practice. A total of 3327 individuals consented to participate and were followed approximately every 18 months for up to nine visits. All assessments were performed by trained physicians and psychologists using standardized questionnaires in the patient's home environment.

The present analyses include 1025 participants 79–94 years of age from follow‐up Wave 3 (FU‐3; 2007–2009), the first wave with available serum samples for PUFA quantification. FU‐3 was designated as baseline (BL) for this study, with up to 8.5 years of follow‐up. Participants with DAT at BL were excluded. All procedures received ethics approval, and written informed consent was obtained. Further details can be found in the Supplementary Information 2.1.

*MAPT dataset*. MAPT enrolled community‐dwelling adults ≥70 years of age meeting at least one of the following: spontaneous memory complaint, impaired instrumental activities of daily living, or slow gait speed.^19^, ^20^, ^21^, ^22^, ^23^ Exclusion criteria included dementia, Mini‐Mental State Examination^24^ (MMSE) <24, impaired basic activities of daily living, or PUFA supplementation at BL.^25^ Of 1679 participants with available 6‐year FU data, 1146 with complete ARA and EPA measures at BL and 1‐year FU were included according to the original intention‐to‐treat framework.

### 2.2 Cognitive assessment outcomes

DAT diagnoses in AgeCoDe adhered to the National Institute of Neurological and Communicative Disorders and Stroke and the Alzheimer's Disease and Related Disorders Association (NINCDS‐ADRDA) criteria for probable AD dementia.^26^ DAT was assessed by consensus of the interviewing investigator and an experienced geriatrician or geriatric psychiatrist according to the Diagnostic and Statistical Manual of Mental Disorders, Fourth Edition (DSM‐IV) and International Classification of Diseases, 10th Revision (ICD‐10) criteria that are implemented as a standardized diagnostic algorithm in the Structured Interview for the Diagnosis of Dementia of Alzheimer type, Multi‐infarct Dementia and Dementias of other Aetiology according to DSM‐IV and ICD‐10 (SIDAM).^27^, ^28^

A cognitive composite *Z*‐score in the MAPT study was generated as described previously,^19^ combining four cognitive tests (further information is available in Supplementary Information 2.2).

### 2.3 Genome‐wide genotyping and association studies

AgeCoDe DNA samples were isolated from peripheral blood using the Qiagen Blood Isolation Kit (Qiagen, Germany). DNA concentration and purity were determined using the NanoDrop ND1000 spectrophotometer (Thermo Fisher Scientific). AgeCoDe cohort samples were genotyped with the Illumina Infinium Global Screening Array (GSA; Illumina, San Diego, CA, USA). Genome‐wide data were not available for MAPT.

Suggestive signals from our PUFA ARA/EPA ratio genome‐wide association study (GWAS) and single nucleotide polymorphisms (SNPs) located within the fatty acid desaturases 1 and 2 (FADS1/FADS2) region, a critical area influencing PUFA metabolism (see Figure S1),^29^, ^30^ were selected for analysis of their association with DAT progression. All quality control (QC) steps for the genetic data from the GSA and the GWAS pipeline are described elsewhere.^31^ Briefly, QC included low call rate, deviations from Hardy‐Weinberg equilibrium, sex discordance, high relatedness, and genetic ancestry outliers. The data were imputed using the TOPMed Imputation Server (Michigan, USA). Genetic variants with minor allele frequency (MAF) ≥0.01 and an imputation quality score (R2) ≥0.30 were used for analysis. Statistical analyses included logistic regression for the ARA/EPA ratio, after correction for sex, age, and three genetic principal components.

### 2.4 Fatty acid profiling

In AgeCoDe, serum phospholipid fatty acids were measured in duplicate using gas chromatography–flame ionization detector (GC‐FID) following established protocols (Shimadzu GmbH, model GC 2010 Plus, Duisburg, Germany; FID).^32^, ^33^

In MAPT, plasma and erythrocyte fatty acids were measured using validated chromatographic methods.^19^

Additional methodological details are available in Supplementary Information 2.3.

### 2.5 Statistical analyses

Analyses were conducted using SPSS 25.0 (SPSS Inc., Chicago, IL) and R 4.0.5, including the *survival* and *lme4* packages for mixed‐effects modeling.^34^, ^35^, ^36^ EPA, ARA, dihomo‐γ‐linolenic acid (DGLA), and the ARA/EPA ratio were expressed as percent composition (see Supplementary Information 2.4 for details). Tertiles of PUFA measures were used for analyses in AgeCoDe. For MAPT, analyses utilized continuous PUFA measures to align with the original publication.^19^ Tertiles were used solely for visualization purposes.

In AgeCoDe, Cox proportional hazards models were used to examine the association between PUFA tertiles and the ARA/EPA ratio with the risk of progressing to DAT, adjusting for age, sex, education, and body mass index (BMI). Likelihood ratio tests were used to evaluate whether the ARA/EPA ratio provided explanatory value beyond EPA alone or beyond EPA and ARA alone. Harrell's concordance index (C‐index) was used to quantify predictive improvement. Additional sensitivity analyses were conducted. Specifically, models including ARA and EPA as independent predictors were fitted and compared using likelihood ratio tests and the Akaike Information Criterion (AIC). To avoid possible reverse causation, another sensitivity analysis was performed by excluding dementia cases in the first year of follow‐up.

Two‐sample Mendelian randomization (MR) analyses were conducted to assess potential causal relationships between genetically determined PUFA ARA/EPA ratio levels and the risk of progression to DAT. Genetic instruments were selected based on results from our PUFA ARA/EPA ratio GWAS in AgeCoDe. Following previously published methods,^37^ SNPs were evaluated using genome‐wide significant (*p* < 5.00 × 10^−8^) and suggestive genome‐wide significance (*p* < 1.00 × 10^−5^) thresholds for further analyses of the outcome.^31^ Instrument strength was assessed using the F‐statistic. Causal estimates were obtained using inverse‐variance weighted (IVW) analysis as the primary method. Sensitivity analyses included MR‐Egger regression to assess directional pleiotropy and the weighted median method to assess robustness to invalid instruments. In addition, functional‐MR analyses were conducted using variants within the *FADS1*/*2* gene region, and a restricted analysis was performed using two key PUFA‐modulation variants identified by linkage disequilibrium (LD) analyses (rs174546 and rs968567; Figure S1). For all MR analyses, SNPs were clumped using an r^2^ threshold of 0.001 to ensure independence.

A linear mixed‐effects model was used in MAPT to examine the association between longitudinal cognitive decline over 6 years and either individual PUFA levels or the ARA/EPA ratio. All models included fixed and random effects for time, PUFA change (either alone or as the ARA/EPA ratio), sex, age at baseline, and intervention group. In addition, the interaction between the time variables and these factors was analyzed. The association of PUFA with cognitive decline was assessed in two ways: (1) first at baseline, including only participants from the placebo group, and (2) by examining changes in the ARA/EPA ratio from baseline to follow‐up during the study.

**FIGURE 2:**
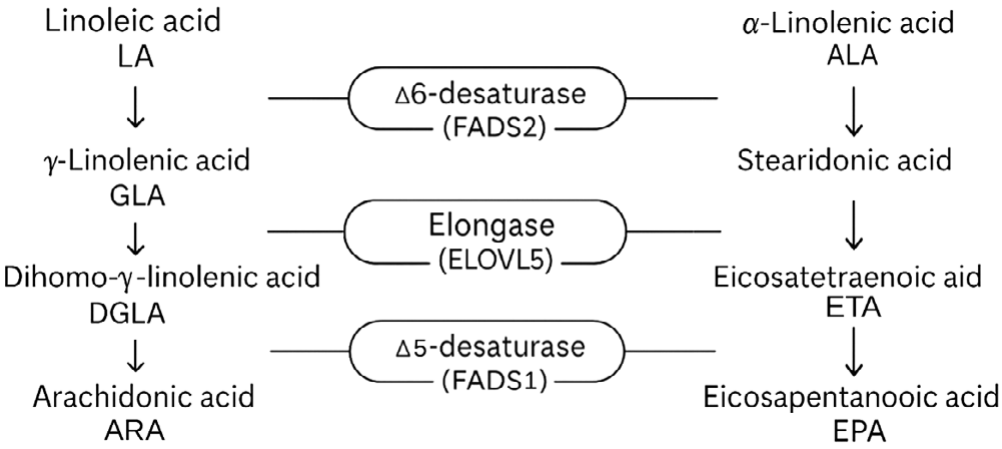
ω6‐PUFA and ω3‐PUFA metabolism pathway. Visualization of some of the main steps on the PUFA pathway. At the same level, pathway metabolites on each branch, that is, ω6‐PUFA or ω3‐PUFA, compete for the same enzymes.

Finally, to examine the biochemical interplay between ω3‐PUFA and ω6‐PUFA species (Figure 2), ARA and DGLA levels were evaluated across EPA tertiles using analysis of variance, followed by Tukey post hoc comparisons. The effects of intervention on this modulation were evaluated at baseline and after 1 year of supplementation.

## 3 RESULTS

### 3.1 ω6‐PUFA/ω3‐PUFA ratio imbalance predicts progression to DAT in AgeCoDe

**FIGURE 3:**
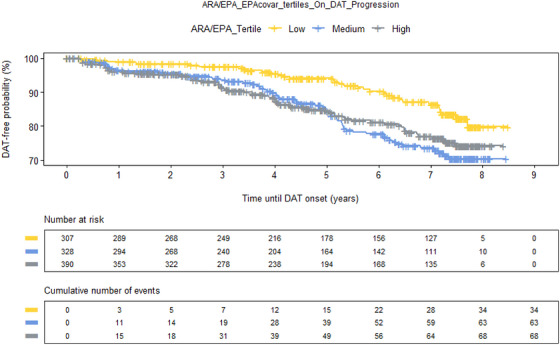
The association between ω6‐PUFA/ω3‐PUFA ratio and the risk for DAT. There is a significant effect of the ARA/EPA ratio on the progression to DAT, indicating an association between a higher baseline ARA/EPA ratio and an increased risk to develop DAT in the AgeCoDe study.

Building on our prior findings on ω3‐PUFA and ω6‐PUFA,^38^, ^39^ we investigated whether the ARA/EPA ratio, as an integrative biomarker of PUFA balance, better captures the risk of progression to DAT than single PUFA levels. Individuals in the higher and middle ARA/EPA tertiles exhibited significantly elevated risk of progressing to DAT relative to the lowest (healthiest) tertile (higher tertile [H_T_]: hazard ratio (HR) = 1.84, 95% confidence interval [CI] 1.21–2.78, *p* = 4.06 × 10^−3^; medium tertile (M_T_): HR = 2.00, 95% CI 1.31–3.03), *p* = 1.19 × 10^−3^; Figure 3, Table 1, Table S2). There was no significantly elevated risk when comparing the higher ARA/EPA tertile to the middle tertile, indicating no linear effect in AgeCoDe (HR = 0.86, 95% CI 0.58–1.28, *p* = 0.46).

**TABLE 1:** Association of PUFA levels and their effect estimates individually or as a ratio in DAT or cognitive decline.

*Association with*	*Cohort*	*Fatty acid ratio*	*HR*	*95% CI*	*Standard error*	*Z‐statistic*	*p*‐value
*AD dementia risk*	AgeCoDe	ARA/EPA mid tertile	1.995	1.314– 3.029	0.213	3.241	1.19x10^−3^
		ARA/EPA high tertile	1.835	1.213–2.776	0.211	2.873	4.06x10^−3^
		ARA mid tertile	0.887	0.610– 1.289	0.191	−0.630	0.53
		ARA high tertile	1.153	0.792‐1.679	0.192	0.741	0.46
		EPA mid tertile	0.666	0.465– 0.956	0.184	−2.206	2.74x10^−2^
		EPA high tertile	0.591	0.401–0.872	0.198	−2.652	8.01x10^−3^

Association with	Cohort	Fatty acid ratio	Time term interaction	Estimate (beta)	Standard error	t‐value	*p*‐value
*Composite cognitive score*	MAPT (at BL)	ARA/EPA	linear	^−^0.094	0.057	−3.035	2.56x10^−3^
		ARA	linear	^−^0.021	0.031	−0.676	0.50
		EPA	linear	0.056	0.031	1.785	7.50x10^−2^
*Composite cognitive score*	MAPT (BL–FU)	ARA/EPA	linear	0.125	0.044	2.823	4.86x10^−3^
			quadratic	^−^0.099	0.062	−1.596	0.11
		ARA	linear	0.192	0.271	0.795	0.43
			quadratic	^−^0.201	0.298	−0.467	0.64
		EPA	linear	^−^0.045	0.055	−0.818	0.41
			quadratic	0.032	0.084	0.384	0.70

Abbreviations: ARA, arachidonic acid; CI, confidence interval; EPA, eicosapentaenoic acid; HR, hazard ratio.

Full details of models results can be found in the supplementary files: Tables S2, S3, S7, and S8.

AgeCoDe models were adjusted using age, sex, BMI, and education.

MAPT has two observation times, and it is also measured according to the difference of baseline and follow‐up points. MAPT, also includes ARA/EPA ratio at baseline as covariate, to control for the effect of the intervention on the change of PUFA levels. MAPT analyses were adjusted by age, sex and intervention group.

In AgeCoDe there is a significant association effect of baseline ARA/EPA and the progression to DAT.

In MAPT there is a significant association effect of baseline ARA/EPA and the composite cognitive *Z*‐score.

In MAPT there is a significant effect of the change of the ARA/EPA ratio interaction with time, indicating its association with the composite cognitive *Z*‐score.

Consistent with prior research,^39^ higher EPA levels were protective (H_T_: HR = 0.59, 95% CI = 0.40–0.87, *p*  = 8.01 × 10^−3^; M_T_: HR = 0.67, 95% CI = 0.47–0.96, *p*  = 2.74 × 10^−2^; Table 1), whereas ARA alone was not significantly associated with the risk of progressing to DAT (H_T_: HR = 1.15, 95% CI = 0.79–1.68, *p*  = 0.46; M_T_: HR = 0.89, 95% CI = 0.61–1.29, *p*  = 0.53; Table 1). When both EPA and the ARA/EPA ratio were included in a single model, the ratio remained a stronger predictor compared to EPA alone (ARA/EPA: H_T_: HR = 1.87, 95% CI = 0.94–3.72, *p*  = 7.59 × 10^−2^; M_T_: HR = 2.42, 95% CI = 1.35–4.34, *p*  = 2.98 × 10^−3^ | EPA: H_T_: HR = 1.01, 95% CI = 0.53–0.92, *p*  = 0.99; M_t_: HR = 0.65, 95% CI = 0.43–0.99, *p*  = 4.56 × 10^−2^; Table S3A). A likelihood ratio test confirmed a statistically significant improvement in the predictive performance when the ARA/EPA ratio was included (*χ* ^2^ = 9.63, degrees of freedom [df] = 2, *p*  = 8.11 × 10^−3^; Table S3A). C‐index comparisons provided similar evidence for added predictive value for the ratio (C‐index_EPA+ARA/EPA _= 0.66, standard error [SE] = 0.21 versus C‐index_EPA _= 0.64, SE = 0.22, *p* = 4.48 × 10^−2^). Our sensitivity analysis, which compared models including ARA and EPA independently, with or without the ARA/EPA ratio, revealed a significant improvement due to the ratio (*χ* ^2^ = 8.78, df = 2, *p* = 1.24 × 10^−2^; Table S3B). In addition, when excluding subjects diagnosed with DAT at the first follow‐up, similar results were observed (*χ* ^2^ = 7.43, df = 2, *p* = 2.43 × 10^−2^; Table S3C).

Direct comparisons between models further confirmed that the ARA/EPA ratio is more predictive than individual PUFA measures. Our earlier analysis using only the ARA/EPA ratio showed stronger associations than the model that included ARA and EPA tertiles separately (AIC_Ratio _= 2085.51; AIC_ARA+EPA _= 2091.56). In that model, only EPA remained significant (EPA: H_T_: HR = 0.59, 95% CI = 0.40–0.87, *p* = 8.09 × 10^−3^; M_T_: HR = 0.65, 95% CI = 0.45–0.94, *p*  = 2.13 × 10^−2^; Table S3B).

### 3.2 Genetic modulation of PUFA levels influences DAT risk

In two‐sample MR analyses using SNPs selected at a suggestive threshold (*p* < 1.00 × 10^−5^), the average F‐statistic (mF) was 19.93, indicating sufficient instrument strength. However, there was no evidence of a causal effect of the genetically predicted ARA/EPA ratio on DAT risk across different MR methods (IVW: *β* = −8.51 × 10^−6^, SE = 5.46 × 10^−4^, *p* = 0.99; weighted median: *β* = 6.45 × 10^−5^, SE = 7.78 × 10^−4^, *p* = 0.93; MR‐Egger: *β* = 6.13 × 10^−6^, SE = 1.00 × 10^−3^, *p* = 0.99; Table S4A). Similar findings were observed with SNPs at a genome‐wide significant threshold (IVW: *β* = −3.34 × 10^−3^, SE = 3.00 × 10^−3^, *p* = 0.27; Table S4B).

Because PUFA metabolism is genetically regulated,^29^, ^30^, ^40^, ^41^ we examined whether SNPs in the *FADS* locus affect the risk of developing DAT. All genetic signals in this region are in strong linkage disequilibrium (LD), suggesting they likely reflect a single genetic influence on PUFA blood levels (see Figure S1).^29^, ^30^, ^40^, ^41^ However, functional MR analyses using variants within the *FADS1*/*2* locus yielded similar non‐significant results (IVW: *β* = 6.39 × 10^−2^, SE = 4.34 × 10^−2^, *p* = 0.14; weighted median: *β* = 6.16 × 10^−2^, SE = 0.14, *p* = 0.66; MR‐Egger: *β* = 2.29 × 10^−2^, SE = 0.10, *p* = 0.84; Table S4C). Nonetheless, the instrument strength for this analysis was weak (mF = 0.11), limiting interpretability. Likewise, analyses focusing only on two key variants (rs174546 and rs968567) did not show significant associations (IVW: *β* = 6.63 × 10^−2^, SE = 6.61 × 10^−2^, *p* = 0.32; Table S4D). Notably, these SNPs are not linked to AD susceptibility according to the latest large‐scale AD GWAS (rs174546: *p* = 0.48 | rs968567: *p* = 0.51).^31^

### 3.3 ω3‐PUFA levels modulate ω6‐PUFA species in blood

**FIGURE 4:**
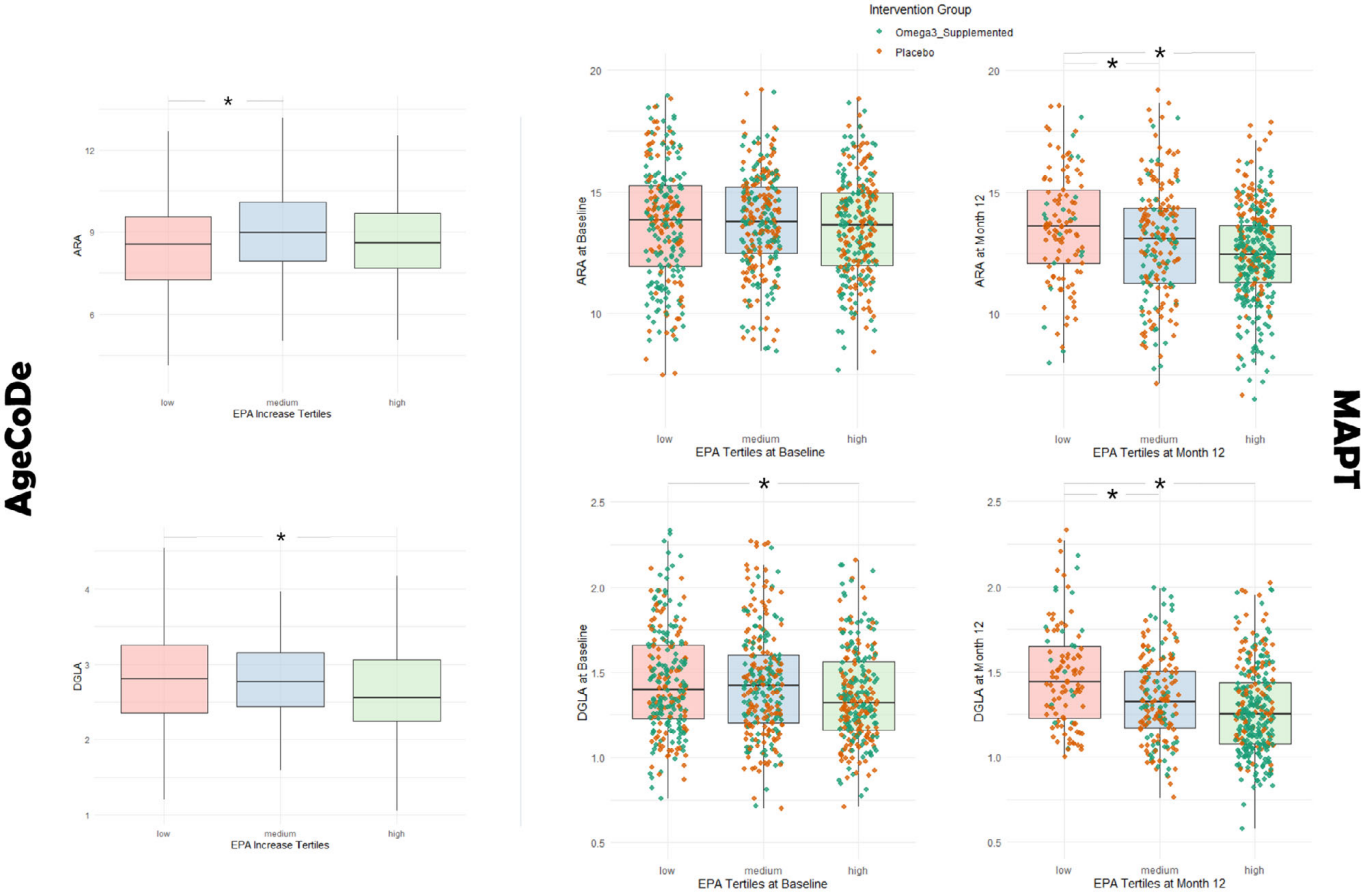
EPA tertile modulation on the levels of the ω6‐PUFA pathway on AgeCoDe and MAPT. There is a significant modulation of higher blood concentrations of the ω3‐PUFA, EPA, on the levels of the ω6‐PUFA species, ARA and DGLA. AgeCoDe is evaluated at BL, whereas MAPT has two observation times, and it is evaluated at BL and FU. The intervention effect of ω3‐PUFA intake as supplement in MAPT increased the effects observed at BL on the ω6‐PUFA metabolites.

The known competition between ω6‐PUFA and ω3‐PUFA for metabolizing enzymes^42^, ^43^, ^44^ prompted us to test whether part of the added value of the ARA/EPA ratio derives from ω3‐PUFA interfering with ω6‐PUFA metabolism. Consequently, we explored the role of the ω3‐PUFA, EPA, in the metabolic step converting DGLA to ARA, which is shared with EPA. Across EPA tertiles, we observed a non‐linear increase in ARA of 7.98% in the medium tertile and 3.42% in the higher tertile of EPA, compared with the lowest tertile of EPA (ARA_mediumEPAtert _= 0.58, 95% CI = 0.28–0.87, *p*‐adj = 0.18; ARA_higherEPAtert _= 0.22, 95% CI = −0.07–0.51, *p*‐adj = 1.30 × 10^−5^; Figure 4, Table S5, Table S6). For DGLA, the ω6‐PUFA precursor of ARA, we observed a dose‐dependent decrease of 1.02% in the medium tertile and 8.30% in the higher tertile of EPA, compared with the lowest tertile of EPA (DGLA_mediumEPAtert _= −0.04, 95% CI = −0.15–0.07, *p*‐adj = 0.69; DGLA_higherEPAtert _= −0.23, 95% CI = −0.33–0.12, *p*‐adj = 3.80 × 10^−6^; Figure 4, Table S5, Table S6). These patterns are consistent with competitive substrate use within the desaturase pathway, underscoring the biochemical interdependence of ω3‐PUFA and ω6‐PUFA metabolism.

### 3.4 Reduced ω6‐PUFA/ω3‐PUFA ratio predicts improved cognitive trajectories in MAPT

To test whether the protective association of a healthier PUFA is reflected in cognitive performance, we examined, in the MAPT intervention study, whether treatment‐related changes in the ARA/EPA ratio were associated with cognitive performance over time. To do so, we compared the mean fold change (FC) from BL to FU in the ARA/EPA ratio and each PUFA alone between the ω3‐PUFA supplementation group and the placebo group. This analysis showed that ω3‐PUFA supplementation led to a 5.90‐FC reduction in the ARA/EPA ratio compared with the placebo group (ARA/EPA_FC _= 5.90, 95% CI = 4.25 to 8.24, *p* = 2.54 × 10^−9^; Figure S2). This was driven by a concomitant decrease in ARA levels of 4.81‐FC in the ω3‐PUFA supplementation group compared with the placebo group (ARA_FC _= 4.81, 95% CI = 0.75 to 1.99, *p* = 1.82 × 10^−5^; Figure S2).

In line with the effect on progression to DAT in AgeCoDe, a higher ARA/EPA ratio at BL predicted faster cognitive decline among participants from the placebo group (*β* = −0.09, SE = 0.06, *p* = 2.56 × 10^−3^; Table 1, Table S7), indicating that each unit increase of the ARA/EPA ratio at baseline was associated with an additional decrease in the cognitive score of 0.09 standard deviation [SD] per year. EPA alone did not show an association with cognitive decline (*β* = 0.06, SE = 0.03, *p* = 7.50 × 10^−2^; Table 1).

**FIGURE 5:**
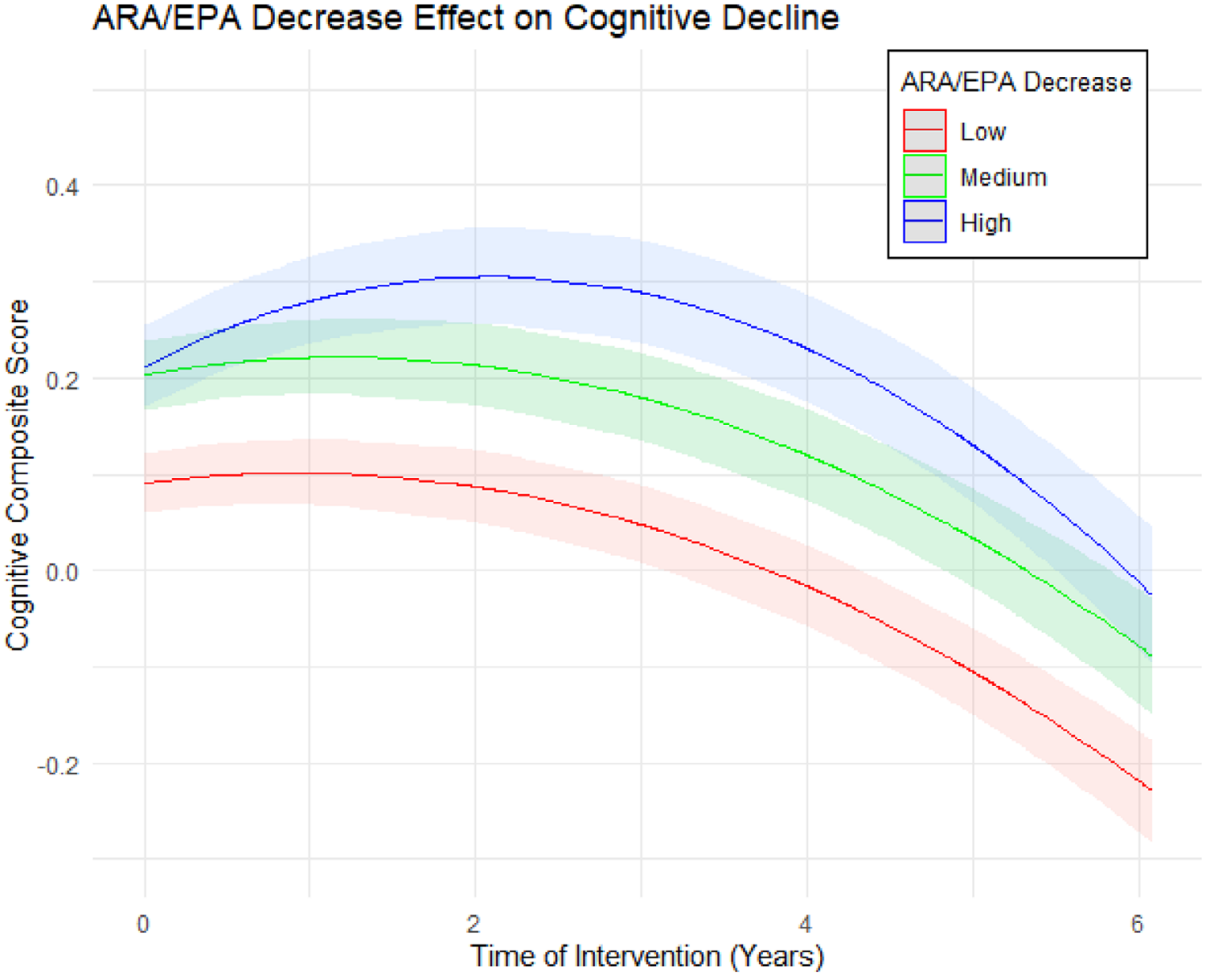
The association of the decrease in ARA/EPA ratio on cognitive decline due to intervention in the MAPT study. There is a significant effect of the decrease in the ARA/EPA ratio on the cognitive outcome during MAPT intervention, indicating an association between the decrease of the ARA/EPA ratio and the longitudinal composite cognitive *Z*‐score in the study. The higher decrease of the ratio after 1 year of intervention follows higher cognitive outcomes and, therefore, less cognitive decline.

Longitudinal analyses showed that individuals who achieved larger reductions in the ARA/EPA ratio during the intervention exhibited significantly greater cognitive improvement over 6 years than those whose ratio remained stable or worsened (time x (ARA/EPA): *β* = 0.13, SE = 0.04, *p* = 4.86 × 10^−3^; (time^2^) x (ARA/EPA): *β* = −0.10, SE = 0.06, *p* = 0.11; Figure 5, Table 1, Table S8). Notably, changes in ARA or EPA alone did not predict cognitive trajectories (ARA: time x ARA: *β* = 0.19, SE = 0.27, *p* = 0.43; (time^2^) x ARA: *β* = −0.20, SE = 0.30, *p* = 0.64; EPA: time x EPA: *β* = −0.05, SE = 0.06, *p* = 0.41; (time^2^) x EPA: *β* = 0.03, SE = 0.08, *p* = 0.70; Table 1). These results were fully replicated using the model structure from the original MAPT publication, ^19^ supporting the robustness of the ratio‐based effects observed in our study (time x (ARA/EPA): *β* = 0.14, SE = 0.05, *p* = 1.69 × 10^−3^; (time^2^) x (ARA/EPA): *β* = −0.39, SE = 0.19, *p* = 4.41 × 10^−2^; (time^3^) x (ARA/EPA): *β* = 0.33, SE = 0.21, *p* = 0.11; Table S9). These cognitive improvements, depending on changes in the ARA/EPA ratio, were not evident in the original analysis, probably because the PUFA balance was not considered in the published results, only isolated PUFA changes.

### 3.5 ω3‐PUFA levels modulate ω6‐PUFA species in MAPT

As in AgeCoDe, EPA levels in MAPT at BL were associated with lower DGLA levels, that is, 0.76% reduction in the medium tertile and 5.75% in the higher tertile of EPA when compared to the lowest tertile (DGLA_mediumEPAtert _= −0.02, 95%CI = −0.08 to 0.05, *p*‐adj = 0.84; DGLA_higherEPAtert _= −0.08, 95% CI = −0.15 to −0.02], *p*‐adj = 4.95 × 10^−6^; Figure 4, Table S5, Table S6). Likewise, ARA levels in MAPT at BL show the same trend of increase along with the increase of EPA levels, with a 0.51% increase in the medium tertile and 1.24% decrease in the higher tertile of EPA when compared to the lowest tertile (ARA_mediumEPAtert _= 0.11, 95% CI = −0.35 to 0.57, *p*‐adj = 0.83; ARA_higherEPAtert _= −0.15, 95% CI = −0.61 to 0.31, *p*‐adj = 0.72; Figure 4, Table S5, Table S6).

We then evaluated these changes after the intervention. For DGLA, we found a 9.77% decrease in the medium tertile and 13.57% decrease in the higher tertile of EPA when compared to the lowest tertile (DGLA_mediumEPAtert _= −0.12, 95% CI = −0.19 to –0.04], *p*‐adj = 7.16 × 10^−4^; DGLA_higherEPAtert _= −0.18, 95% CI = −0.25 to –0.12, *p*‐adj = 1.00 × 10^−8^; Figure 4, Table S5, Table S6). Notably, ARA exhibits a larger change after intervention, showing a 5.24% decrease in the medium tertile and 8.27% in the higher tertile of EPA when compared to the lowest one (ARA_mediumEPAtert _= −0.70, 95% CI = −1.30 to –0.10, *p*‐adj = 1.65 × 10^−2^; ARA_higherEPAtert _= −1.10, 95% CI = −1.65 to –0.55, *p*‐adj = 9.20 × 10^−6^; Figure 4, Table S5, Table S6).

Given this reduction in ARA after intervention, we finally aimed to explore whether the decrease in ARA itself during intervention was linked to cognitive decline. Our findings showed that individuals receiving ω3‐PUFA supplementation, who had the most significant reduction in ARA, experienced less cognitive decline over six years (time x ARA_decrease: *β* = 0.14 [SE = 0.06], *p* = 1.48 × 10^−2^; (time^2^) x ARA_decrease: *β* = −0.16 [SE = 0.08], *p* = 3.60 × 10^−2^; Figure S3, Table S10).

## 4 DISCUSSION

Cognitive impairment and dementia result from multiple interacting factors, including modifiable ones like nutrition. In two independent cohorts, we show that the balance between ω6‐PUFA and ω3‐PUFA is more strongly associated with cognitive decline and progression to DAT than the levels of either PUFA type alone. Furthermore, circulating ω3‐PUFA, particularly EPA, appears to regulate ω6‐PUFA species and their metabolism. Over time, this regulation seems to reduce ω6‐PUFA levels and improve the overall ω6‐PUFA/ω3‐PUFA balance. Overall, these findings indicate that dietary approaches focused on balancing PUFA by adjusting both ω6‐PUFA and ω3‐PUFA could be more effective for preserving cognitive health than merely taking ω3‐PUFA supplements.

Previous intervention studies focusing solely on ω3‐PUFA have produced inconsistent results regarding cognition and dementia risk.^7^, ^8^, ^9^, ^10^, ^19^, ^45^, ^46^, ^47^ These interventions typically supplement both EPA and DHA. In our study, we focused on EPA for two reasons: previous research in AgeCoDe indicated that only EPA shows a consistent association with the risk of progression to DAT,^39^ and EPA was more relevant to the ratio used because it shares the same metabolic step with ARA and competes for the same FADS enzyme. In addition, the ARA/EPA ratio is a recognized biomarker of PUFA balance associated with cardiovascular disease and type 2 diabetes risk.^4^, ^44^ However, the role of ω6‐PUFA, particularly ARA, in dementia remains unclear, despite emerging evidence linking it to increased dementia risk.^48^, ^49^, ^50^, ^51^ Our findings now indicate that the interplay between ω6‐PUFA and ω3‐PUFA metabolism may be more influential on cognitive outcomes and progression to DAT than either pathway branch alone. Of note, our results support prior research showing that a lower (healthier) ARA/EPA ratio is associated with a decreased risk of progressing to DAT.^4^, ^44^ Moreover, re‐analysis of MAPT data further highlights the importance of ratio changes, indicating that greater increases in the ratio toward healthier values during the intervention correlate with improved cognitive outcomes. These larger ratio shifts were driven primarily by reductions in ω6‐PUFA levels among MAPT participants receiving ω3‐PUFA supplements. Notably, these participants experienced the greatest cognitive protection after the intervention, with the largest decreases in ω6‐PUFA levels and the greatest improvements in the ARA/EPA ratio.

These results highlight the metabolic interaction between ω6‐PUFA and ω3‐PUFA pathways, which depend on shared desaturase and elongase enzymes.^13^, ^42^, ^43^, ^52^ These enzymes typically prefer ω3‐PUFA substrates. However, under a WD with excess ω6‐PUFA, the enzymes are primarily engaged in processing ω6‐PUFA. In MAPT, the most significant changes in the ARA/EPA ratio occurred in the ω3‐PUFA supplement group. These changes are likely due to two factors: greater EPA availability after supplementation and the role of EPA in decreasing ω6‐PUFA levels. EPA may also shift enzyme preference toward ω3‐PUFA and displace ARA in cell membranes, thereby reducing the production of pro‐inflammatory mediators.^53^, ^54^, ^55^ Participants who showed the most significant improvement in the ARA/EPA ratio also experienced the most notable cognitive benefits, underscoring the importance of this biomarker for overall health and cognition.

Our findings add to the emerging evidence that PUFA balance may influence the clinical progression of AD. However, two‐sample MR found no evidence that genetically determined ARA/EPA ratio causally affects the risk of progressing to DAT across several methods, including inverse‐variance weighted, weighted median, and MR‐Egger. Functional MR analyses focusing on variants within the *FADS1/2* locus, as well as analyses based on key variants (rs174546 and rs968567), also showed consistent null results. Notably, neither rs174546 nor rs968567 shows an association with AD susceptibility in the latest large‐scale GWAS meta‐analysis of AD,^31^ suggesting that PUFA‐related biology is unlikely to contribute to the initial development of AD pathology. Instead, the effect appears to be restricted to downstream mechanisms that shape how rapidly symptoms progress once underlying pathology is present. This distinction between factors influencing disease onset versus progression carries important clinical implications. Our results suggest that interventions aimed at modifying PUFA balance, particularly dietary approaches targeting the ω6‐PUFA/ω3‐PUFA ratio, may hold greater promise for slowing progression rather than preventing disease onset.

The exact biological mechanisms linking PUFA balance to cognitive decline and dementia remain debated, but they likely involve age‐related cognitive decline due to increased brain vulnerability to both neurodegenerative and other processes.^13^, ^56^, ^57^ ω6‐PUFA and ω3‐PUFA support membrane fluidity, synaptic plasticity, inflammation control, and cardiovascular health.^58^ Imbalances may lead to neuroinflammation, synaptic loss, and impaired learning and memory.^59^ Maintaining a healthier ω6‐PUFA/ω3‐PUFA ratio might reduce these issues by modulating microglial activity^58^ and immune responses to abnormal proteins, such as amyloid,^59^ potentially increasing neuronal survival and cognitive function.^60^ Further research is necessary, including longitudinal intervention studies, to understand the mechanisms driving these effects and the dietary factors that can influence PUFA balance and, consequently, disease progression in meaningful ways.

Our study has both strengths and limitations. Strengths include the use of two independent European cohorts with complementary observational and interventional designs, longitudinal cognitive assessments, and harmonized PUFA profiling. The consistent associations observed in both AgeCoDe and MAPT enhance the predictive reliability of the ω6‐PUFA/ω3‐PUFA ratio. Because the AgeCoDe study involves older adults who regularly visit primary care, its findings may directly inform public health recommendations. Limitations include reliance on serum phospholipids in AgeCoDe.^61^ PUFA levels in plasma/serum phospholipids mainly reflect relatively recent dietary fatty acid intake, that is, within the past few days and weeks, whereas concentrations in blood cells, such as erythrocytes, are considered better indicators of long‐term status, including dietary intake and endogenous conversion. Finally, this composition may differ from tissue‐ or blood cell–based PUFA pools.^62^, ^63^, ^64^ Despite thorough adjustment for confounders, residual confounding remains possible. However, PUFA measured in plasma or serum phospholipids has frequently been used in epidemiological studies, and fatty acid profiles of serum/plasma phospholipids have been shown to correlate with erythrocyte membrane phospholipid fatty acid levels.^32^, ^65^ One additional limitation concerns our genetic instruments for the ARA/EPA ratio, which are based on a relatively small GWAS, potentially weakening their power and reducing statistical strength. In addition, although we cannot completely rule out some sample overlap between the exposure and outcome GWAS, its effect is likely minimal based on our findings.

In conclusion, maintaining a healthier ω6‐PUFA/ω3‐PUFA ratio, by reducing ω‐6 PUFA intake and increasing consumption of fatty fish and other ω3‐PUFA–rich sources, may help preserve cognitive function, slow cognitive decline, and reduce the risk of progression to DAT. The ARA/EPA ratio emerges as a practical biomarker for monitoring both dietary adherence and metabolic response. Of note, current dietary recommendations and intervention strategies have focused largely on increasing ω3‐PUFA intake. Although our findings support diet as the most effective and modifiable means of influencing PUFA balance, they also indicate that increasing ω3‐PUFA intake alone may be insufficient to meaningfully shift the ratio. Instead, our results emphasize that improving the ω6‐PUFA/ω3‐PUFA balance likely requires a combined approach, in which moderation of ω6‐PUFA intake is combined with increased ω3‐PUFA intake to achieve more substantial effects on disease progression.

Consequently, future preventive strategies and dietary interventions should prioritize optimizing the ω6‐PUFA/ω3‐PUFA ratio rather than focusing solely on ω3‐PUFA supplementation or individual PUFA species. Such an approach may offer greater potential to delay progression to DAT and mitigate the long‐term public health burden associated with dementia.

## CONFLICT OF INTEREST STATEMENT

All authors declare no conflicts of interest. Author disclosures are available in the Supporting Information.

## CONSENT STATEMENT

All human subjects provided informed consent.

## Supporting information



Supporting Information

Supporting Information

Supporting Information

Supporting Information

Supporting Information

Supporting Information

Supporting Information

Supporting Information

Supporting Information

Supporting Information

Supporting Information

Supporting Information

Supporting Information

Supporting Information

Supporting Information
