# Supplementary material for: Prediction of cognitive outcome and progression to dementia using ω6‐PUFA/ω3‐PUFA ratio
Source: Alzheimers Dement. 2026 Jun 10;22(6):e71590. doi: 10.1002/alz.71590 (PMC13253362; doi:10.1002/alz.71590)
Supplement: Supplementary file 1 — Supporting Information [file ALZ-22-e71590-s004.docx]

**Supplementary Table 1.** **Summary of the AgeCoDe study and MAPT intervention cohorts.**

| **Dataset** | **Characteristics** | **Non-converter (n=860)** | | **AD Dementia (n=165)** | | **P-value** |
| --- | --- | --- | --- | --- | --- | --- |
| **AgeCoDe** | Age [years] | 84.3 ± 0.11 | | 85.5 ± 0.26 | | 8.6x10^-5^ |
|  | Female [%] | 64.0 | | 73.3 | | 2.6x10^-2^ |
|  | BMI | 26.0 ± 0.14 | | 25.3 ± 0.29 | | 4.6x10^-2^ |
|  | CASMIN  [% high] | 14.0 | | 12.1 | | NS |
|  | 20:4ω-6  ARA [%] | 9.11 ± 2.02 | | 9.20 ± 1.81 | | NS |
|  | 20:5ω-3  EPA [%] | 1.02 ± 0.46 | | 0.92 ± 0.42 | | 7.0x10^-3^ |
|  | 20:4ω-6 / 20:5ω-3 ratio (ARA/EPA) | 10.43 ± 5.25 | | 11.89 ± 5.74 | | 1.0x10^-3^ |
| **Dataset** | **Characteristics** | **Placebo (n=283)** | **Placebo**  **+MI (n=294)** | **ω3-PUFA**  **(n=290)** | **ω3-PUFA**  **+MI (n=279)** | **P-value** |
| **MAPT** | Age [years] | 75.0 ± 0.26 | 75.1 ± 0.24 | 75.3 ± 0.26 | 75.5 ± 0.28 | NS |
|  | Female [%] | 67.8 | 63.6 | 60.0 | 63.4 | NS |
|  | 20:4ω-6  ARA [%] | 13.5 ± 0.13 | 13.7 ± 0.13 | 13.5 ± 0.12 | 13.5 ± 0.14 | NS |
|  | 20:5ω-3  EPA [%] | 1.00 ± 0.025 | 1.00 ± 0.025 | 0.96 ± 0.028 | 0.94 ± 0.024 | NS |
|  | 20:4ω-6 / 20:5ω-3 ratio (ARA/EPA) | 15.9 ± 0.43 | 16.4 ± 0.49 | 17.2 ± 0.52 | 17.1 ± 0.51 | NS |
| Abbreviations: ω-3, omega-3 fatty acid; ω-6, omega-6 fatty acid; ARA, arachidonic acid; EPA, eicosapentaenoic acid; NS, not significant; student’s t-test for continuous variables, Pearson χ² test for categorical variables. | | | | | | |
