# Supplementary material for: Prediction of cognitive outcome and progression to dementia using ω6‐PUFA/ω3‐PUFA ratio
Source: Alzheimers Dement. 2026 Jun 10;22(6):e71590. doi: 10.1002/alz.71590 (PMC13253362; doi:10.1002/alz.71590)
Supplement: Supplementary file 5 — Supporting Information [file ALZ-22-e71590-s011.docx]

**Supplementary Table 5. EPA tertiles modulation on the levels of the ω6-PUFA pathway on AgeCoDe and MAPT.** There is a significant modulation effect of higher EPA levels in blood, on the levels of the ω6-PUFA species ARA, and its precursor, DGLA. AgeCoDe is evaluated at BL, while MAPT has two observation times, and it is evaluated at BL or FU. The intervention effect of ω3-PUFA intake as supplement in MAPT increased the effects observed on the ω6-PUFA branch, i.e. DGLA and ARA metabolites.

**
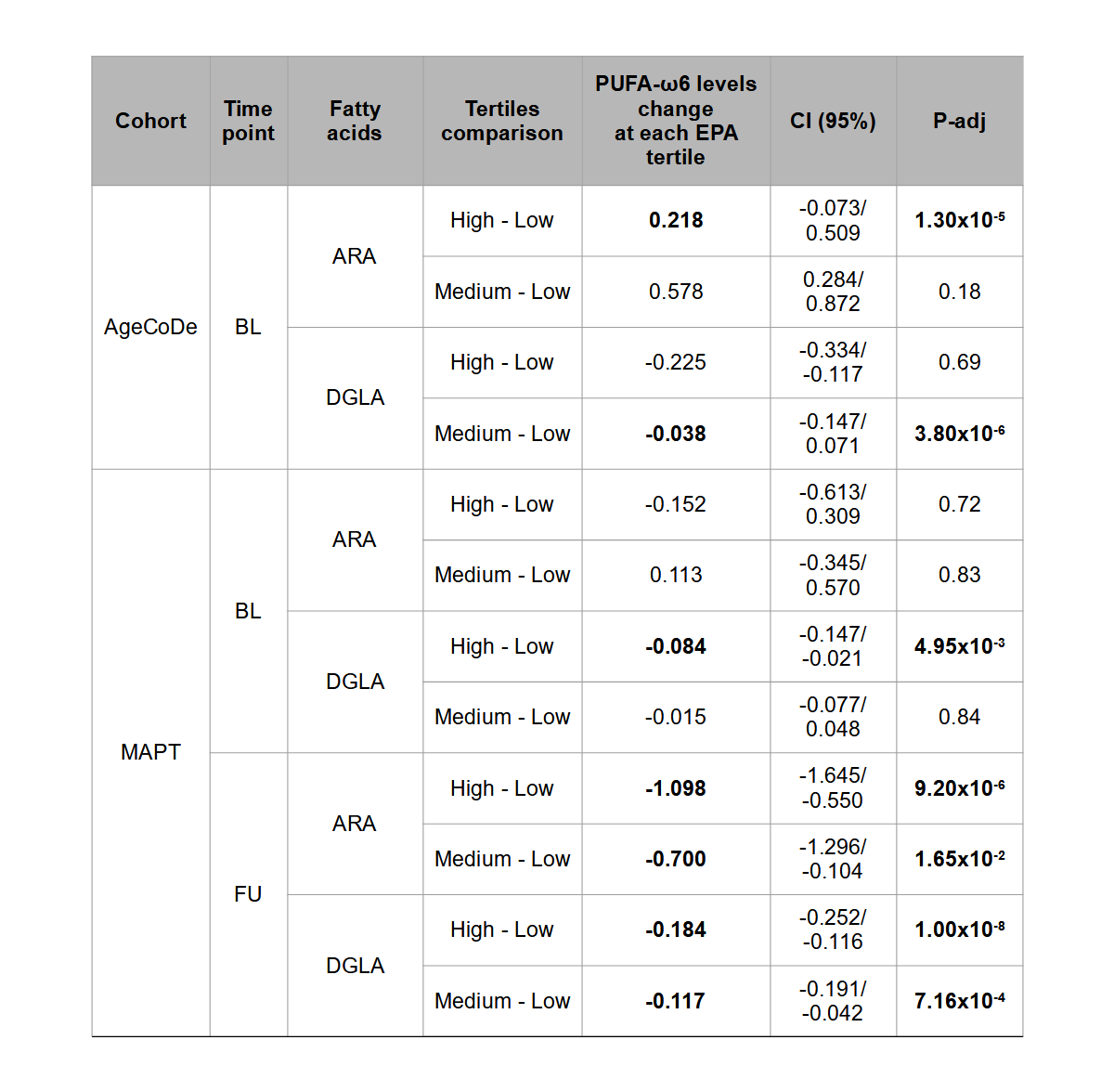
**
