# Supplementary material for: Prediction of cognitive outcome and progression to dementia using ω6‐PUFA/ω3‐PUFA ratio
Source: Alzheimers Dement. 2026 Jun 10;22(6):e71590. doi: 10.1002/alz.71590 (PMC13253362; doi:10.1002/alz.71590)
Supplement: Supplementary file 11 — Supporting Information [file ALZ-22-e71590-s001.docx]

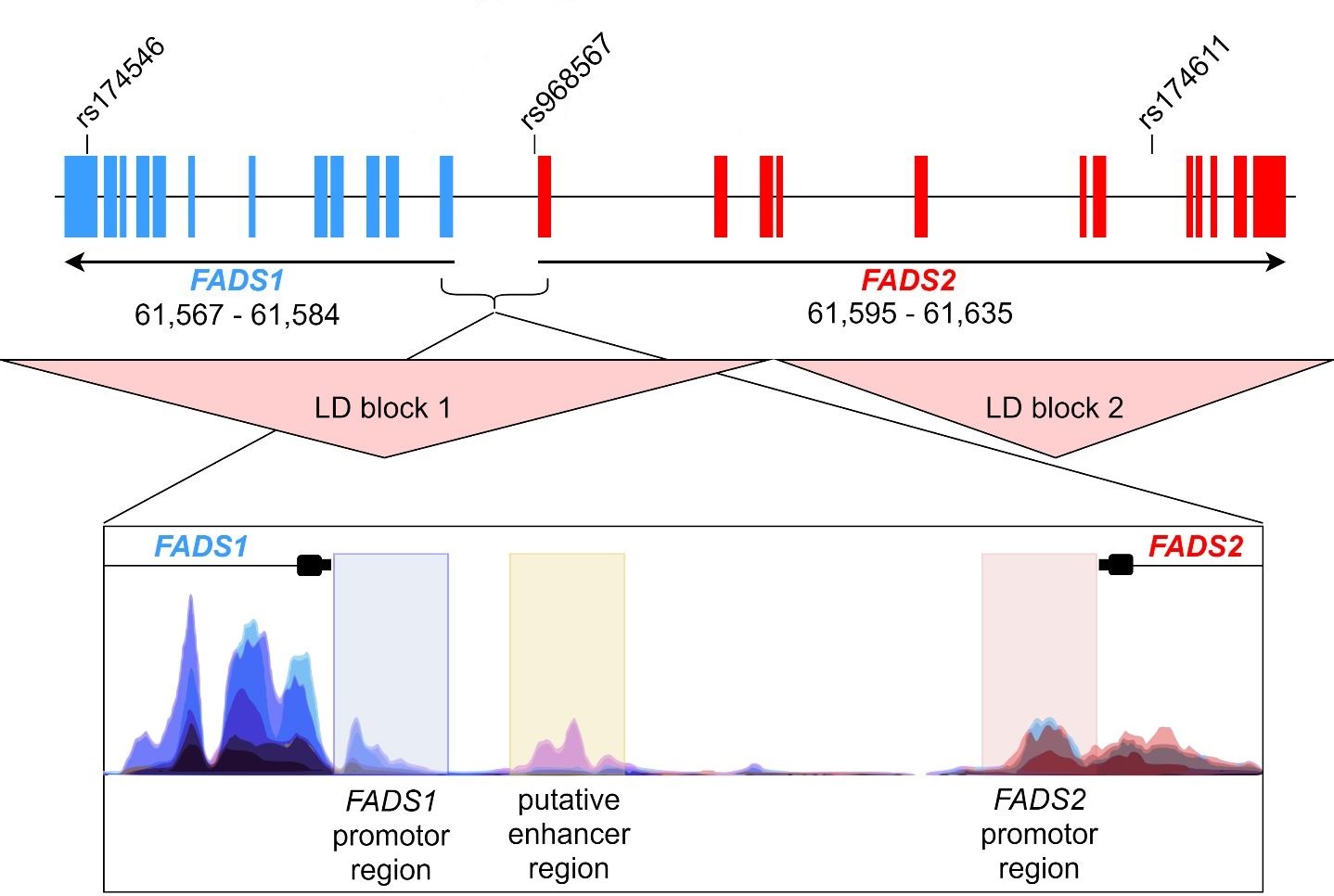


**Supplementary Figure 1.** ***FADS1/2* gene cluster.** The SNPs used for the association analysis of the effects of PUFA levels on the risk of progression to DAT, are marked in the upper illustration. The haploblocks are indicated by LD blocks 1 and 2. The lower illustration highlights the key regulatory regions depicted by the H3K27Ac cell line from the ENCODE project^*^ between the *FADS1/2* genes with the *FADS1* promotor region (blue), *FADS2* promotor region (red), and the putative enhancer region (yellow). *ENCODE Project Consortium. An integrated encyclopedia of DNA elements in the human genome. *Nature*. 2012;489(7414):57-74.
