# Supplementary material for: Prediction of cognitive outcome and progression to dementia using ω6‐PUFA/ω3‐PUFA ratio
Source: Alzheimers Dement. 2026 Jun 10;22(6):e71590. doi: 10.1002/alz.71590 (PMC13253362; doi:10.1002/alz.71590)
Supplement: Supplementary file 12 — Supporting Information [file ALZ-22-e71590-s005.docx]

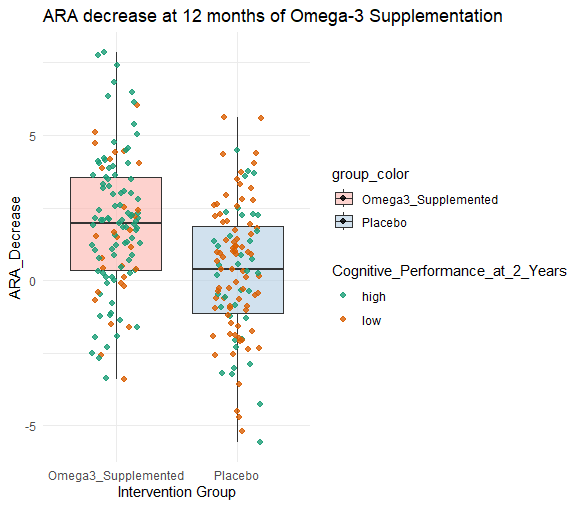

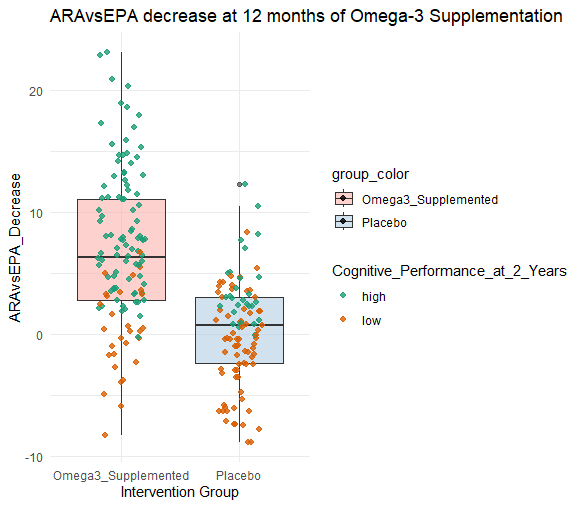

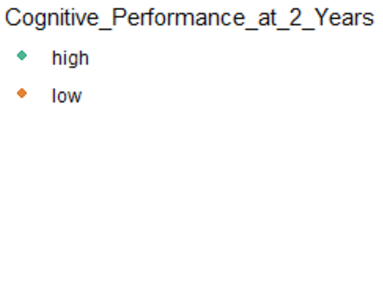

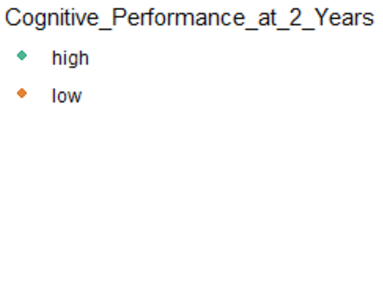


**Supplementary Figure 2. ARA/EPA ratio and ARA individual levels change due to the supplementation of ω3-PUFA.** A significant effect of those individuals who received ω3-PUFA supplements as intervention (groups *ω3-PUFA* and *ω3-PUFA+MI*) can be observed in comparison to those who did not receive ω3-PUFA during their intervention (groups *Placebo* and *MI*) in the MAPT study. The levels of ARA and ARA/EPA decreased the most at 1 year of intervention in the groups receiving ω3-PUFA as supplement in comparison with those who received placebo. Cognitive performance is evaluated using the mean cognitive composite score at 2 years as threshold for high or low levels. **MI: Multidomain Intervention*.
