# Supplementary material for: Prediction of cognitive outcome and progression to dementia using ω6‐PUFA/ω3‐PUFA ratio
Source: Alzheimers Dement. 2026 Jun 10;22(6):e71590. doi: 10.1002/alz.71590 (PMC13253362; doi:10.1002/alz.71590)
Supplement: Supplementary file 13 — Supporting Information [file ALZ-22-e71590-s014.docx]

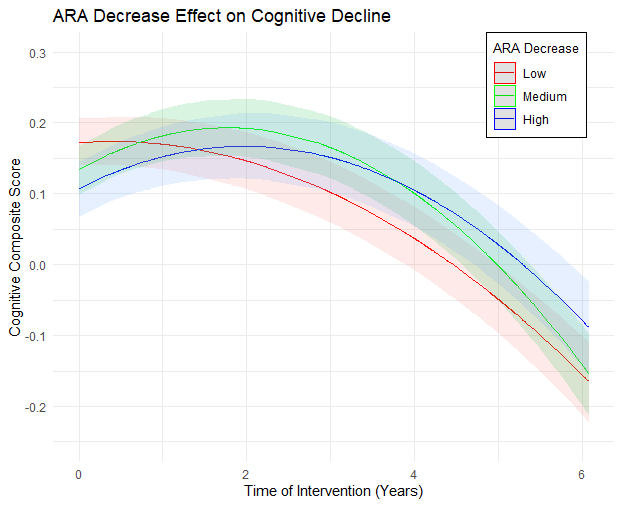


**Supplementary Figure 3. The association of the decrease in ARA on the cognitive decline due to intervention in the MAPT study.** There is a significant effect of the decrease in ω6-PUFA, ARA, on the cognitive outcome during MAPT intervention, indicating an association between the decrease of ARA and the longitudinal composite cognitive Z-score in the study. The higher decrease of ARA after one year of intervention, follows higher cognitive outcomes and therefore, less cognitive decline.
