# Supplementary material for: Prediction of cognitive outcome and progression to dementia using ω6‐PUFA/ω3‐PUFA ratio
Source: Alzheimers Dement. 2026 Jun 10;22(6):e71590. doi: 10.1002/alz.71590 (PMC13253362; doi:10.1002/alz.71590)
Supplement: Supplementary file 14 — Supporting Information [file ALZ-22-e71590-s002.docx]

**Supplementary Material**

**Methods**

2.1.Data sets and study design

*The AgeCoDe dataset.* The AgeCoDe study is a general practice (GP) registry-based longitudinal study of subjects recruited at 75 years and above in six German cities from 2003 to 2004^1^. Exclusion criteria were consultations only by home visits by the GP, residence in a nursing home, severe illness the GP would deem fatal within 3 months, insufficient facility in German, deafness or blindness, lacking the ability to consent, and not being a regular patient of the participating practice. A total of 3327 patients gave informed consent for participation and received follow-up assessments every 18 months. All assessments were performed by trained physicians and psychologists using standardized questionnaires in the patient’s home environment. Time point of PUFA measurement (FU-3) was used as the new analysis baseline (collected between 2007-2009). Sample measurements used for the analyses can be found at Figure 1.

*The MAPT dataset.* The MAPT study^2^ included community-dwelling participants with 70 years or older that met at least one of three criteria: spontaneous memory complaint expressed to their physician, limitation in one instrumental activity of daily living^3^, or slow gait speed (≤0.8 m/s, or more than 5 s to walk 4 m)^4-6^. Participants with a Mini-Mental State Examination^7^ (MMSE) score lower than 24, those in whom dementia was diagnosed, and those with any difficulty in basic activities of daily living^8^ were excluded, as were those taking polyunsaturated fatty acid supplements at baseline. From 1679 patients with 6 years of follow-up (FU) information, all individuals with ARA and EPA PUFA measurements at BL and FU were considered for further analyses (n = 1149; as shown on Figure 1).

The respective ethics committees (AgeCoDe: Local ethics committees at Hamburg, Bonn, Düsseldorf, Heidelberg/Mannheim, Leipzig, Munich. | MAPT: French Ethical Committee in Toulouse [Comité de Protection des Personnes/CPP SOOM II].) approved the present study and written informed consent was obtained from all participants before inclusion. All study procedures complied with national legislation and the Code of Ethical Principles for Medical Research Involving Human Subjects of the World Medical Association, in accordance with the ethical standards in the 1964 Declaration of Helsinki and its later amendments.

2.2.Cognitive assessment and diagnostic criteria

*AgeCoDe Study*. Cognitive function and dementia were assessed by consensus of the interviewing investigator and an experienced geriatrician or geriatric psychiatrist according to the Diagnostic and Statistical Manual of Mental Disorders 4th Edition (DSM-IV) and International Classification of Diseases (ICD-10) criteria that are implemented as a standardized diagnostic algorithm in the Structured Interview for Diagnosis of Dementia of Alzheimer type, Multi-infarct Dementia and Dementia of other Aetiology according to DSM-IV and ICD-10 (SIDAM)^9,10^.

The SIDAM is specifically designed to diagnose dementia, comprising of cognitive impairment, defined by the total SIDAM cognitive score (SIDAM cognitive (SISCO) score)^7^. The diagnosis of dementia in AD was established according to the National Institute of Neurological and Communicative Disorders and Stroke and the Alzheimer’s Disease and Related Disorders Association (NINCDS-ADRDA) criteria for probable AD dementia^11^.

Dementia diagnosis in participants who were not personally interviewed was based on the Global Deterioration Scale (GDS)^12^ and the Blessed Dementia Rating (BDR) scale^13^. A score of at least four on the GDS was used as the diagnostic criteria for dementia. In these cases, an etiological diagnosis was established if the information provided was sufficient to judge etiology according to the aforementioned criteria.

*MAPT Study*. The cognitive performance was evaluated as previously described^2^. The primary efficacy outcome was a change from baseline to 72 months in a composite Z-score combining four cognitive tests, i) free and total recall of the Free and Cued Selective Reminding Test^14^, ii) ten MMSE orientation items, iii) the Digit Symbol Substitution Test score from the Wechsler Adult Intelligence Scale-Revised^15^, and iv) the Category Naming Test^16^ (ie, 2 min category fluency in animals).

2.3.Fatty acid profiling

*AgeCoDe.* The fatty acid (FA) composition of serum phospholipids was determined in duplicate by gas chromatography. Briefly, serum samples were defrosted at room temperature, and an internal standard was added. Total lipids were extracted using methanol:chloroform according to a modified Folch method^17^. The phospholipid fraction was separated using thin-layer chromatography according to the method of Christophe and Matthijs^18^ using a silica thin-layer chromatography plate in a solvent mixture of petroleum ether and acetic acid. After scraping off the phospholipid band under ultraviolet light, the phospholipid fraction was methylated by transesterification with methanol/HCl and incubated at 95°C for 4 h. The FA methyl esters were extracted with petroleum ether, dissolved in heptane, and injected into the gas chromatograph. Peaks of interest were identified by comparing with authentic FA methyl ester standards (37 Component FAME Mix certified reference material, C14-C24, Sigma-Aldrich, St. Louis, MI, USA). Selected FAs were expressed as a percentage of the total area by dividing the integrated area under the peak by the total area of all FAs.

*MAPT.* Samples were obtained from plasma and erythrocytes. These were initially extracted utilizing isopropanol and hexane, followed by transmethylation with methanol and sulfuric acid. Subsequent processing involved evaporation and redissolution in isooctane prior to chromatographic analysis^19^.

2.4.Statistical analyses

The proportion of an individual FA was calculated accordingly to formula (1).

%single FA in PUFA = 100 × single FA / FA_measured_ (1)

In the AgeCoDe sample, 37 FA were considered for the percentage calculation, meanwhile, the MAPT sample FA percentages were calculated with 51 available FA. To determine in MAPT the effects of the interventions with supplementation of ω3-PUFA against the placebo groups, an analysis of variance was performed followed by a Tukey multiple comparisons of means for the different EPA tertile effects over DGLA and ARA. Two-sample Student’s t-test was employed to evaluate the ARA/EPA ratio decrease, and ARA individual decrease during the intervention, between the ω3-PUFA and placebo groups.

**References**

1. Luck T, Riedel-Heller SG, Kaduszkiewicz H et al. Mild Cognitive Impairment in General Practice: Age-Specific Prevalence and Correlate Results from the German Study on Ageing, Cognition, and Dementia in Primary Care Patients (AgeCoDe). *Dement Geriatr Cogn Disord.* 2007;24(4):307-316.

2. Andrieu, S. et al. Effect of long-term omega 3 polyunsaturated fatty acid supplementation with or without multidomain intervention on cognitive function in elderly adults with memory complaints (MAPT): a randomised, placebo-controlled trial. *Lancet Neurol.* 2017;16:377-389.

3. Lawton, MP. Scales to measure competence in everyday activities. *Psychopharmacol Bull.* 1988;609-14.

4. Alfaro-Acha, A., et al. Does 8-foot walk time predict cognitive decline in older Mexicans Americans? *J Am Geriatr Soc.* 2007;55:245-51.

5. Abellan van Kan, G., et al. Gait speed at usual pace as a predictor of adverse outcomes in community-dwelling older people an International Academy on Nutrition and Aging (IANA) Task Force. *J Nutr Health Aging.* 2009;13:881-89.

6. Studenski, S. et al. Gait speed and survival in older adults. JAMA; 305: 50–58 (2011).

7. Folstein, MF., et al. “Mini-mental state”. A practical method for grading the cognitive state of patients for the clinician. J Psychiatr Res; 12: 189–98 (1975).

8. Katz, S., et al. Studies of illness in the aged. The index of ADL: a standardized measure of biological and psychosocial function. *JAMA*. 1963;185:914-19.

9. Zaudig M, Mittelhammer J, Hiller W, et al. SIDAM–A structured interview for the diagnosis of dementia of the Alzheimer type, multi-infarct dementia and dementias of other aetiology according to ICD10 and DSM-III-R. *Psychol Med.* 1991;21(1):225-236.

10. Zaudig M., Hiller W. SIDAM-Handbuch Strukturiertes Interview Für Die Diagnose Einer Demenz vom Alzheimer Typ, der Multiinfarkt- (Oder Vaskulären) Demenz und Demenzen anderer Ätiologie nach DSM-III-R, DSM-IV, ICD-10. Hans Huber; Bern, Switzerland: (1996).

11. McKhann, G., et al. Clinical diagnosis of Alzheimer’s disease: report of the NINCDS-ADRDA Work Group under the auspices of Department of Health and Human Services Task Force on Alzheimer’s Disease. *Neurology.* 1984;34(7):pp.939-944.

12. Reisberg B., Ferris S.H., Deleon M.J., Crook T. The Global Deterioration Scale for Assessment of Primary Degenerative Dementia. *Am J Psychiat.* 1982;139:1136-1139.

13. Blessed G. The association between quantitative measures of dementia and of senile change in the cerebral grey matter of elderly subjects-Retrospective. *Int J Geriatr Psych.* 1996;11:1036-1038.

14. Grober E, Buschke H, Crystal H, Bang S, Dresner R. Screening for dementia by memory testing. *Neurology.* 1988;38:900-03.

15. Wechsler D. Wechsler adult intelligence scale-revised. New York: Psychological Corp. (1981).

16. Cardebat D, Doyon B, Puel M, Goulet P, Joanette Y. Formal and semantic lexical evocation in normal subjects. Performance and dynamics of production as a function of sex, age and educational level. *Acta Neurol Belg.* 1990;90:207-17 (in French).

17. Folch J., Lees M., Sloane Stanley G.H. A simple method for the isolation and purification of total lipides from animal tissues. *J Biol Chem.* 1957;226:497-509.

18. Christophe A., Matthijs F. New method for the determination of the fatty acid pattern of serum lipid classes. *Clin Chim Acta.* 1967;16:39-43.

19. Sun Q, Ma J, Campos H, Hankinson SE, Hu FB. Comparison between plasma and erythrocyte fatty acid content as biomarkers of fatty acid intake in US women. *Am J Clin Nutr.* 2007;86:pp.74-81.
